# Unique lignin modifications pattern the nucleation of silica in sorghum endodermis

**DOI:** 10.1093/jxb/eraa127

**Published:** 2020-03-09

**Authors:** Nerya Zexer, Rivka Elbaum

**Affiliations:** 1 The Robert H. Smith Institute of Plant Sciences and Genetics in Agriculture, The Hebrew University of Jerusalem, Rehovot, Israel; 2 Comenius University, Slovakia

**Keywords:** Endodermis, lignin, root, silica, silicic acid, silicon, *Sorghum bicolor*

## Abstract

Silicon dioxide in the form of hydrated silica is a component of plant tissues that can constitute several percent by dry weight in certain taxa. Nonetheless, the mechanism of plant silica formation is mostly unknown. Silicon (Si) is taken up from the soil by roots in the form of monosilicic acid molecules. The silicic acid is carried in the xylem and subsequently polymerizes in target sites to silica. In roots of sorghum (*Sorghum bicolor*), silica aggregates form in an orderly pattern along the inner tangential cell walls of endodermis cells. Using Raman microspectroscopy, autofluorescence, and scanning electron microscopy, we investigated the structure and composition of developing aggregates in roots of sorghum seedlings. Putative silica aggregation loci were identified in roots grown under Si starvation. These micrometer-scale spots were constructed of tightly packed modified lignin, and nucleated trace concentrations of silicic acid. Substantial variation in cell wall autofluorescence between Si+ and Si– roots demonstrated the impact of Si on cell wall chemistry. We propose that in Si– roots, the modified lignin cross-linked into the cell wall and lost its ability to nucleate silica. In Si+ roots, silica polymerized on the modified lignin and altered its structure. Our work demonstrates a high degree of control over lignin and silica deposition in cell walls.

## Teaser

Silica deposition in roots of sorghum is highly controlled. Modified lignin is deposited in a spotted pattern along the cell walls of endodermis cells. These spots act as silica nucleation sites, leading to the formation of silica aggregates.

## Introduction

Silicon (Si) is a highly abundant mineral in specific plant tissues, yet its contribution to plant biology is poorly understood. It is well known that stressed plants lacking access to silicic acid suffer in many respects (Ma and Yamaji, 2006; Coskun *et al.*, 2019). Therefore, although Si is not an essential element, it is extremely important. Si is present in most soils as various forms of Si dioxide, constituting the major environment of plant roots (Currie and Perry, 2007). Soil silicates dissolve and release monosilicic acid [Si(OH)_4_], which is available for root plant uptake. The silicic acid moves with water in the plant to target locations, mostly in the shoot epidermis. There, the acid deposits as solid hydrated silica (SiO_2_·nH_2_O), also called biogenic opal or silica (herein referred to as silica). The current paradigm is that the solid biomineral has little interaction with plant biochemistry because it is insoluble after deposition (Yoshida *et al.*, 1962). Its effects are thus limited to its material properties. Some examples are reduction of cell wall digestibility (Van Soest and Jones, 1968; Massey and Hartley, 2009), fortification of prickles and hairs (Bauer *et al.*, 2011; Mustafa *et al.*, 2018), and filtering of UV light (Goto *et al.*, 2003; Pierantoni *et al.*, 2017). However, exposing plants to soluble silicic acid improves their performance under a wide variety of stresses (Epstein, 2009; Liang *et al.*, 2015). The mechanism by which Si (as silica or silicic acid) influences plant physiology remains elusive (Detmann *et al.*, 2012; Vivancos *et al.*, 2015; Markovich *et al.*, 2017; Coskun *et al.*, 2019).

Plants may take up Si passively or actively through channels and transporters termed Low silicon (Lsi1, Lsi2; Ma *et al*., 2006, 2007; Ma and Yamaji, 2015; Coskun *et al.*, 2019). These proteins allow the passage of Si across the endodermis, which otherwise constitutes an impermeable barrier to Si diffusion (Ma and Yamaji, 2015). The Si is then translocated to the shoot via the xylem transpiration stream (Jones *et al.*, 1963). The amount of silica deposited usually correlates with the transpiration rates of an organ (Jones *et al.*, 1963; Trembath-Reichert *et al.*, 2015; McLarnon *et al.*, 2017), suggesting that silicic acid is mostly present in the apoplastic volume. Transpiration is also regarded as the main driving force for the deposition of silica. However, we and others have shown that leaf silicification in silica cells is not interrupted even in the absence of transpiration, and that silicification occurs only in live cells (Sangster and Parry, 1971; Kumar *et al.*, 2017; Kumar and Elbaum, 2018). Sorghum (*Sorghum bicolor* (L.) Moench) silica cells express the silica-depositing protein Siliplant1 (Slp1) and export it to the cell wall, timed with cell mineralization. There, Slp1 interacts with the silicic acid in the apoplast, causing local silica precipitation (Kumar *et al.*, 2019, Preprint).

Silica may deposit within cellulosic cell walls (Hodson, 2016). Cell wall components interacting with silica may include cellulose (Perry and Lu, 1992), hemicelluloses (Perry *et al.*, 1987; He *et al.*, 2015), callose (Brugiére and Exley, 2017; Kulich *et al.*, 2018), lignin (Fang and Ma, 2006), and ferulic acid (Soukup *et al.*, 2017). Silicified structures are frequently associated with lignified tissues (Zhang *et al.*, 2013; Fleck *et al.*, 2015). Silica in rice straw is associated mostly with Klason extracted lignin, constituting ~80% of the total silica (Pan *et al.*, 2017). Several studies have observed lignin–silica trade-offs in plants grown in Si-available soils or media (Goto *et al.*, 2003; Schoelynck *et al.*, 2010; Yamamoto *et al.*, 2012).

**Fig. 1.**
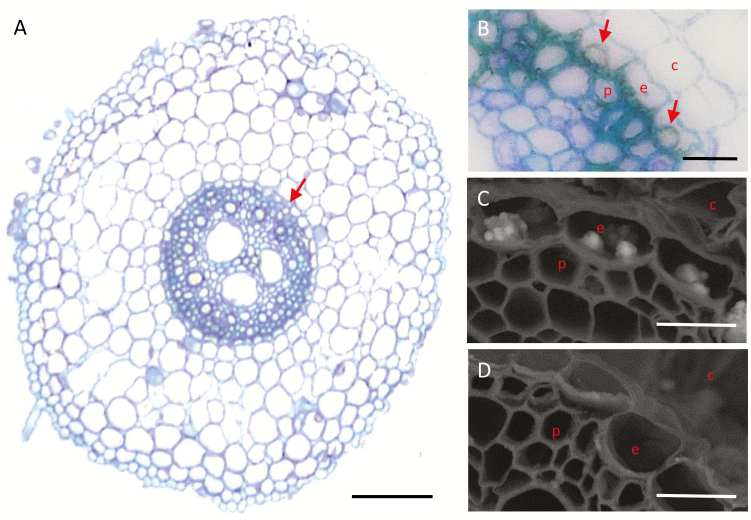
Cross sections of primary roots of sorghum. (A) Light micrograph of a cross section of a Si+ root stained with toluidine blue. (B) Close-up of the endodermal area marked by an arrow in panel A. Silica aggregates (red arrows) form on the ITCW of endodermis cells. (C, D) Scanning electron micrographs in back-scattered electrons mode (SEM-BSE) of the endodermis of (C) Si+ and (D) Si– roots. Silica aggregates (white in appearance) form only in the ITCW of endodermis cells of Si+ roots. c, cortex; e, endodermis, p, pericycle. Scale bars: A=50 µm, B–D=10 µm.

Considerable silicification occurs in the root endodermis of sorghum, in the inner tangential cell wall (ITCW) (Parry and Kelso, 1975; Soukup *et al.*, 2017) (Fig. 1). The deposits, which are internal to the Casparian strips, precipitate from the apoplastic sap of the stele. They are constituted of silicic acid molecules that were taken up by the root transporters and reached the transpiration stream. However, the Si did not flow up to the shoot, but was bound to the thick cell wall at the boundary of the stele. The silica aggregates can form in detached root segments cultivated in silicic acid solution *ex planta*. The mineral forms without any transpiration, in an energy-dependent metabolic process (Soukup *et al.*, 2019).

Partial acid digestion of sorghum roots leaves ferulic acid and hemicellulose associated with the silica aggregates, suggesting that these components are occluded in the mineral (Soukup *et al.*, 2017). Blue fluorescence is associated with intact aggregates, which increases under high pH (Soukup *et al.*, 2014). This enhancement in the fluorescence indicates the content of lignin associated with the mineral (Harris and Hartley, 1976). In grasses, ferulic acid is a common lignin monomer (Ralph *et al.*, 2008) that may also crosslink hemicellulose. An indication of ferulic acid bound to hemicellulose may come from a shift from blue fluorescence under neutral pH to green fluorescence under higher pH (Harris and Hartley, 1976). This shift occurs as a result of ionization of the phenolic hydroxyl of ferulic acid (Rudall and Caddick, 1994). In this work we examine the first stages of silica deposition at the endodermis of sorghum roots. Using fluorescence microscopy combined with Raman microspectroscopy and scanning electron microscopy (SEM), we show that very local lignin modifications induce silica nucleation. These loci are poor in ferulic acid in comparison to their surroundings.

## Materials and methods

### Plant material and growing conditions

Grains of *Sorghum bicolor* (L.) Moench, line BTx623, were surface-sterilized with 3% sodium hypochlorite for 10 min and washed twice with distilled water. Grains were then placed in petri dishes lined with wet filter papers and germinated for 72 h. After germination, seedlings were grown hydroponically for 1 week. All growth solutions were based on double-distilled water containing 1 mM CaCl_2_. Si+ medium was supplied with sodium silicate (Na_2_SiO_3_) at a final concentration of 2 mM. Si– medium was provided with NaCl at a similar final concentration in order to maintain equal ionic balance across all media. The final pH of all the solutions was adjusted to 5.8 with HCl. Seedlings were grown on plates of polystyrene foam mounted on top of darkened 1 litre beakers containing growing media, with 10 seedlings per beaker. Cultivation was done in a growth chamber under controlled conditions, with a photoperiod of 16 h:8 h (light:dark), illuminated with photosynthetically active radiation of approximately 200 μmol m^−2^ s^−1^, 28 °C:22 °C (light:dark) temperature, and 70% air humidity.

### Root tissue embedding and sectioning for light microscopy

Complete primary roots were harvested and fixed in FAA [50% ethanol, 35% double-distilled water, 10% formaldehyde (37%), 5% glacial acetic acid; v/v]. The roots were then divided into four zones: zone i, lowest 4 cm from the root tip; zone ii: 4–8 cm from the root tip; zone iii, 8–12 cm from root tip; zone iv, 12–16 cm from the root tip. Root pieces 5 mm in length were taken from zone iii and embedded using a Leica Historesin embedding kit (Leica, Germany) according to the manufacturer’s instructions. Sections 4 µm thick were obtained using a tungsten carbide blade mounted on a Leica RM2165 microtome. Sections were stained with 0.1% (w/v) toluidine blue for 2 min and washed three times in double-distilled water. Specimens were visualized under a Leica DM500 light microscope equipped with a Leica ICC50W camera (Leica, Germany).

### Scanning electron microscopy and energy-dispersive X-ray analyses

Complete primary roots were harvested and fixed in ethanol:acetic acid (9:1 v/v) under vacuum for 24 hours. Root samples were kept in the same fixative at 4 °C for an additional 48 hours. The fixing solution was then replaced with 70% ethanol and samples were stored at 4 °C until use. The roots were divided into the abovementioned four zones. Cross sections cut by hand were collected from all zones and mounted on aluminum stubs using carbon tape. Other root sections were carefully peeled out of their outer cortical tissues using finely serrated tweezers. Representative segments from all zones were mounted on aluminum stubs using carbon tape. Observations and scanning electron microscopy–energy-dispersive X-ray (SEM-EDX) analyses were performed with a JEOL JSM-IT 100 InTouchScope^TM^ scanning electron microscope (JEOL, Japan) under a low vacuum (30 Pa) and with an accelerating voltage of 20 kV. All micrographs were taken using the back-scattered electrons (BSE) mode (SEM-BSE). SEM-EDX analysis maps were collected at the K-edge using the JEOL integral detector with a dwell time of 0.1 ms and sweep count of 30.

### Confocal and fluorescence microscopy

Peeled root segments were prepared as described for SEM analysis. Root sections 20 mm in length were mounted on glass microscope slides. Samples were immersed in double-distilled water and covered with cover slips. Observations and images were made using a Leica TCS SP8 confocal laser scanning microscope equipped with a ×63 water-immersed objective. The excitation wavelengths used were 405 nm and 488 nm; emission was collected at 400–500 nm and 500–550 nm, respectively. Epifluorescence and bright-field micrographs were collected by a Nikon Eclipse 80i microscope (Nikon, Japan); an X-cite 120Q (Lumen Dynamics, Canada) UV light source was used. Specimens were observed with the following set of Nikon filters: GFP (excitation: 450–490 nm; emission: 500–550) and DAPI (excitation: 400–418 nm; 450–465 nm). Images were taken with a CRI camera controlled by Abrio 1.4 software (PerkinElmer, USA).

### Raman microspectroscopic observation of the endodermal cell wall

Peeled root segments were prepared as described for SEM analysis. Root sections were mounted on aluminum microscope slides using super glue. In cases where roots were chemically treated, root segments were gently shaken for 1 h in 1 M NaOH or 3% acidefid NaOCl (pH 4.5). The samples were then washed tree times with double-distilled water and mounted on aluminium slides. The samples were covered with a drop of double-distilled water and measurements were made using a ×63 water-immersed objective. Raman maps were collected with a Renishaw InVia spectrometer equipped with a 532 nm laser (45 mW maximum intensity, 2 μm^2^ beam), utilizing WiRE3.2 software (Renishaw, New Mills, UK). Measurements were performed using the Streamline mode with acquisition time of 30 s. Spectral analysis was done in WiRE3.2 (Renishaw), including normalization of the total signal to 1000 arbitrary scattering units, background subtraction, and peak picking.

### Co-localization of Raman, SEM, and fluorescence signal maps

Raman maps of single endodermis cells were measured from the edge of a root segment extracted from zone iii. The areas measured were also photographed using the instrument’s internal reflected light microscope. For same-cell SEM-BSE analysis, a measured root segment was carefully transferred from the Raman aluminum slide onto a SEM aluminum stub covered by carbon tape and observed by SEM-BSE. For fluorescence observation of the Raman-mapped cells, the same root segment, still mounted on the aluminum slide was examined. The root segment was immersed in double-distilled water, covered with a cover slip, and imaged by epifluorescence. Identification of the single cells mapped with Raman spectroscopy in SEM-BSE and epifluorescence was achieved by superimposing and aligning the images using ImageJ software (Bethesda, MD, USA).

## Results

Silica aggregates in the endodermis of sorghum roots are associated with a tertiary lignified cell wall that forms after the primary and secondary cell walls have completed their development (Sangster and Parry, 1976) (Fig. 1A, B). These can be identified by SEM-BSE in roots grown hydroponically with silicic acid (Si+). In this scanning mode, Si atoms scatter more electrons than carbon (C) and oxygen (O) atoms, and thus appear brighter than the organic tissue (Fig. 1C). We did not observe silica aggregates in roots grown without silicic acid (Si–) (Fig. 1D).

**Fig. 2.**
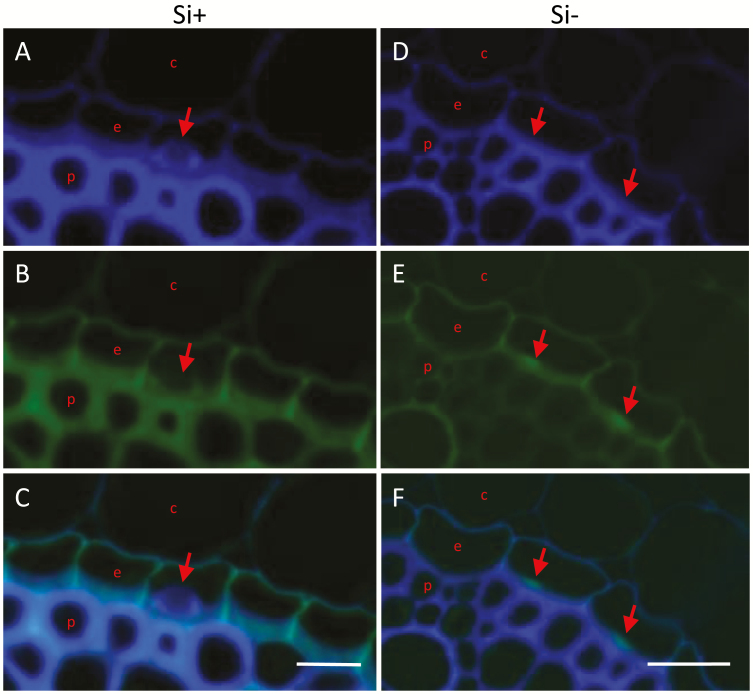
Autofluorescence emitted by cross sections of sorghum roots. (A) Blue, (B) green, and (C) merged image of both signals in Si+ roots. Arrows indicate silica aggregates. The blue fluorescence is more intense at the base and edges of a silica aggregate than in the cell wall itself and at the tip of the aggregate. Green fluorescence of the ITCW outlines the aggregate. (D) Blue, (E) green, and (F) merged image of both signals in Si– roots. Arrows mark the location of spots of green fluorescence in the ITCW. c, cortex; e, endodermis; p, pericycle. Scale bars=10 µm.

In order to study lignin deposition, we examined the autofluorescence of the cross sections (Fig. 2). The ITCW of endodermis cells of Si+ roots exhibited blue fluorescence localized to the rims and base of the aggregate. Green autofluorescence was identified surrounding the silica body (Fig. 2A–C). In contrast, in Si– ITCW we observed a distinct green spot at the center of the cell wall. This feature was not identified in cross sections of Si+ roots (Fig. 2D–F).

**Fig. 3.**
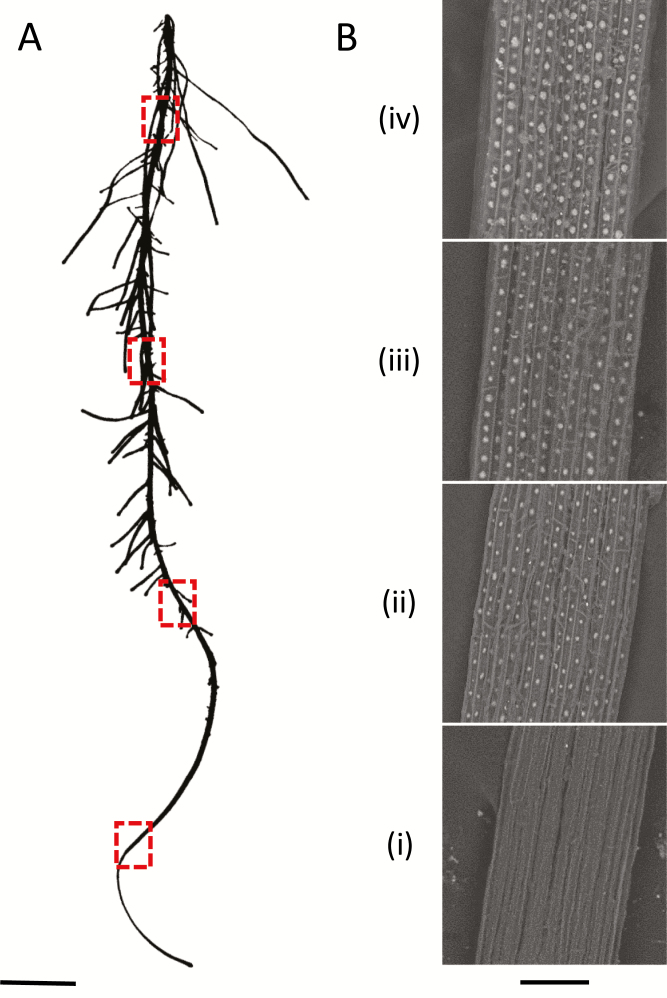
Identification of the active silicification zone (ASZ) in roots of sorghum seedlings. (A) Representative image of a primary root of a 10-day-old sorghum seedling cultivated in Si+ hydroponic solution. Red rectangles indicate the regions observed in panel B. Scale bar=1 cm. (B) To visualize the endodermis along the root, the cortical tissues were removed. SEM-BSE micrographs reveal the silica aggregates (white in appearance). Four distinct silicification regions were defined, starting at the young zone close to the root tip (bottom): (i) no silica aggregation; (ii) aggregates begin to form from approximately 4 cm above the root tip; (iii) aggregates grow in diameter; (iv) at the root base close to the root–shoot junction, new aggregates appear between developed ones. Scale bar=50 µm.

To assess the developmental relationship between silica aggregation and the fluorescence pattern, we defined root zones 4 cm in length, starting at the root tip, which is the youngest part of the root (Fig. 3A). To identify the active silicification zone (ASZ) along roots of Si+ seedlings, we gently stripped roots from their cortex and exposed the developing endodermis. Using SEM-BSE, we identified silica aggregation at 4 cm above the root tip (Fig. 3B, zone ii). The silica aggregates increased in diameter along the developmental gradient of the root. Closer to the root–shoot junction, additional aggregates appeared between more developed ones, filling the ITCW with aggregates (Fig. 3B, zone iv).

**Fig. 4.**
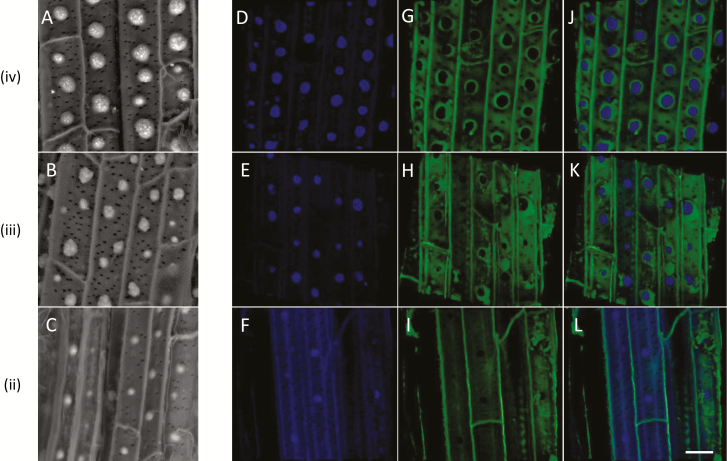
Autofluorescence and SEM of the ITCW of the endodermis, imaged along roots of Si+ sorghum seedlings after removal of the cortical tissues. SEM-BSE micrographs of zones iv (A), iii (B), and ii (C), as defined in Fig. 3. Blue autofluorescence of zones iv (D), iii (E), and ii (F) creates a similar pattern to that of the aggregates. Green autofluorescence of the same samples in zones iv (G), iii (H), and ii (I) is limited mostly to longitudinal cell walls in zone ii; in zones iii and iv, fluorescence is extended to the ITCW, with the exception of non-fluorescent spots encircled by highly fluorescing green rings. (J–L) Merged view of the blue and green autofluorescence images confirms that the green fluorescence delineates the blue fluorescent spots. SEM-BSE and confocal micrographs are independent and do not represent the same cells. Scale bar=10 µm.

A close-up of the silica aggregates in zones ii–iv shows that they form a pattern independent of the numerous pits (seen as black dots in Fig. 4A–C). In zone iii (Fig. 4B), we identified the initiation of small aggregates between more developed ones. In this region, silica deposition seems to be most intensive. Timed with the appearance of the aggregates, we detected a pattern of blue autofluorescent spots (Fig. 4D–F). The spatial distribution of the blue fluorescence was similar to the silicification pattern, suggesting that phenolic molecules are distributed in a similar pattern to the silica. Green fluorescence was detected mostly in the radial cell walls of the young root, in zone ii (Fig. 4I). In zones iii and iv, the fluorescence was also detected in the ITCW (Fig. 4G, H). Interestingly, green fluorescing rings circled the blue fluorescent spots (Fig. 4J, K). This complements our observations in the cross sections (Fig. 2B, C), suggesting that a ring of green fluorescence delineates the silica aggregates at the surface of the ITCW.

**Fig. 5.**
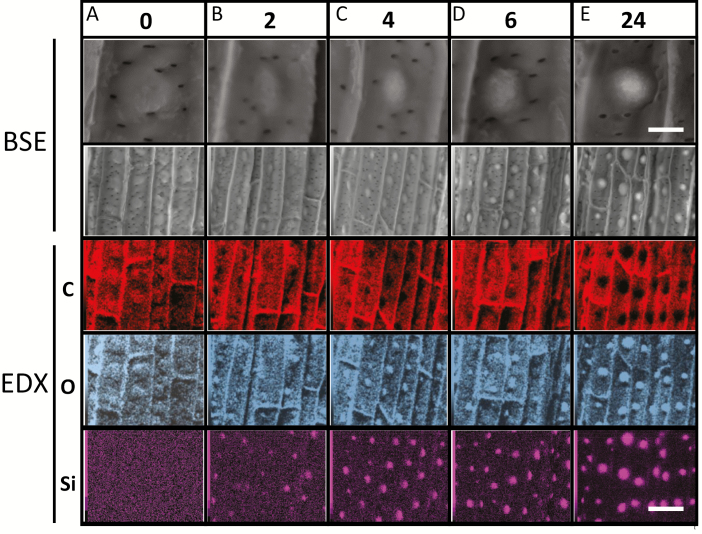
SEM-BSE and EDX imaging of the ITCW of root endodermis in zone iii. Sorghum seedlings were cultivated in Si– medium for 1 week and then transplanted into Si+ medium. Samples were imaged by SEM-BSE at higher (top row) and lower (second row) magnification, and by SEM-EDX for carbon (C; red panels), oxygen (O; gray panels), and silicon (Si; pink panels). (A) Before exposure to Si+ medium (time 0), bright structures were identified by SEM-BSE (top two panels). These structures were richer in C and O, but not in Si. (B) After exposure to Si+ for 2 h, sporadic Si aggregation was detected by SEM-BSE (top two panels) and SEM-EDX. The Si-rich regions were rich in O and poor in C. (C) After exposure for 4 h, the density of the aggregates increased. (D) At 6 h exposure, the aggregates became larger. SEM-BSE at higher magnification (top panel) revealed that the aggregates started to project out of the ITCW. (E) At 24 h exposure, the aggregates were even larger and protruded more from the cell wall, while their density was similar to that at 4 h exposure to Si+ medium. Scale bar in E top panel, common to all top panels, represents 5 µm. Scale bar in E bottom panel, common to all but the top panels, represents 25 µm.

To study the time course of silicification, we exposed Si– roots to Si+ growth solution, and imaged zone iii by SEM-EDX (Fig. 5). At time 0, Si– roots exhibited bright ring-like structures arranged in a pattern that resembled the silica aggregation. Importantly, no Si signal could be measured by SEM-EDX in these spots, and we detected no significant topography related to the spots (see Supplementary Fig. S1 at *JXB* online). The higher BSE signal was attributed to higher signals of C and O atoms, suggesting some differences in cell wall composition and density (Fig. 5A). Initial silica aggregation in distinct spots was detected after 2 h of exposure to Si+ media by both SEM-BSE and SEM-EDX (Fig. 5B). The density of the aggregates was similar to that detected in zone ii (Fig. 4C). At this stage, the silica was mostly embedded within the ITCW. After 4 h of exposure to Si+ solution, we detected small aggregates more or less equidistant from larger ones (Fig. 5C). The locations with high Si exhibited high O and low C, as we would expect with the formation of silica (SiO_2_). With 6 h of exposure, many of the larger aggregates protruded from the ITCW (Fig. 5D). After 24 h, the density of the aggregates remained similar to that at the 4 h exposure (Fig. 5E), and similar to that detected in Si+ grown roots (Fig. 4A). A similar rate of Si deposition was previously reported by Lux *et al*. (2003).

**Fig. 6.**
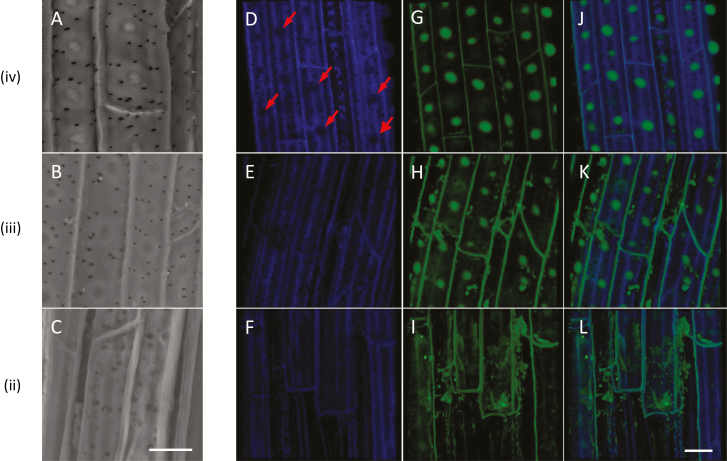
Autofluorescence and SEM of the ITCW of the endodermis, imaged along roots of Si– sorghum seedlings after removal of the cortical tissues. (A–C) SEM-BSE micrographs of zones iv (A), iii (B), and ii (C), as defined in Fig. 3. (D) Blue autofluorescence of zone iv creates a pattern of gaps (red arrows), suggestive of the silica distribution in Si+ roots. The blue autofluorescence pattern in zones iii (E) and ii (F) is irregular, with lines running along the endodermis ITCW long axis, in addition to the fluorescence of the radial cell walls. The pattern of green autofluorescence of the same sample in zone iv (G) complements the blue autofluorescence, creating spots that are distributed similarly to the silica aggregates in Si+ roots. This pattern is initiated in zone iii (H). (I) The green autofluorescence in zone ii is limited mostly to the radial cell walls. A merged image of the blue and green autofluorescence images in zone iv (J) confirms that the green fluorescent spots do not fluoresce in blue. Merged images of the blue and green autofluorescence images of zone iii (K) and zone ii (L) show that the green and blue autofluorescence are mostly separated, except for the radial cell walls, which show both green and blue autofluorescence. SEM-BSE and confocal micrographs are independent and do not represent the same cells. Scale bar=10 µm.

We hypothesized that the non-silicified bright structures we detected in Si–deprived roots (Fig. 5A) are predictors for silica deposition. We thus aimed to study the chemistry of these structures. We first examined the autofluorescence and SEM-BSE-visible features along Si– roots (Fig. 6). The bright cell wall structures visible by SEM-BSE could be detected in zones iii and iv but not zone ii (Fig. 6A–C). Interestingly, we could not detect the blue spotted pattern that characterized the silica aggregation in Si+ roots (compare Fig. 6D–F with Fig. 4). Instead, green autofluorescent spots were apparent in zones iii and iv (Fig. 6G–I). These features can explain the localized green autofluorescence detected in cross sections of Si– root (Fig. 2E). Together, the results of our autofluorescence study suggest that in Si– ITCW a green fluorescent spot lies on top of a blue fluorescent cell wall.

**Fig. 7.**
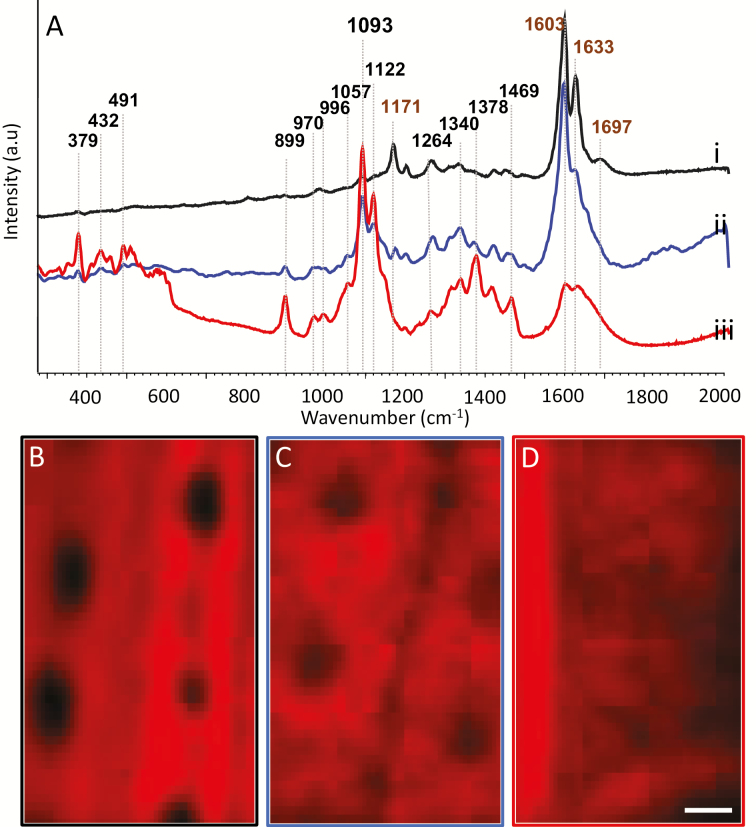
Raman microspectroscopy of the ITCW of root endodermis at zone iii in Si– sorghum seedlings. (A) Spectra of the native ITCW (i) and the cell wall after extraction with 1 M NaOH (ii) or acidic bleach (iii). Peaks were assigned according to Agarwal (2014). Specifically, peaks were identified at the strongest scattering of lignin (at 1171, 1603, 1633, and 1697 cm^−1^) and xylan (at 491 and 1091 cm^−1^), and the strong and medium scattering peaks of cellulose (at 379, 432, 460, 520, 899, 970, 996, 1057, 1096, 1122, 1152, 1294, 1340, and 1378 cm^−1^) and glucomannan (at 1089, 1121, 1264, 1374, and 1469 cm^−1^). Brown text represents lignin residues and black text represents cellulose/hemicellulose residues. The ITCW Raman map of the peak intensity at 1630 cm^−1^, assigned to ferulic acid, was plotted for (B) native ITCW, (C) ITCW treated by NaOH, and (D) ITCW treated by acidic bleach. A spotted pattern of low intensity at 1630 cm^−1^ was detected in the native and NaOH-treated cell walls, but absent in the cell walls treated with acidic bleach. Scale bar=5 µm.

We further studied the chemistry of the Si– ITCW by Raman microspectroscopy and chemical manipulation (Fig. 7). A spectrum typical of lignocellulose was collected from the native ITCW (Fig. 7A, i). We assigned the major scatterings at 1697, 1633, 1603, and 1171 cm^−1^ to lignin and aromatic molecules (Agarwal, 2014). We then treated the ITCW with a solution of 1 M NaOH (Fig. 7A, ii). Hemicelluloses are extracted with this treatment (Carpita, 1984). We found that the typical ferulic acid peaks at 1633 and 1171 cm^−1^ were reduced relative to the total aromatic scattering at 1603 cm^−1^ (Ram *et al.*, 2003). Peaks assigned to cellulose, with the strongest scattering at 1122 and 1093 cm^−1^ (Agarwal, 2014), were smaller in relation to the band representing total aromatics at 1603 cm^−1^. We also examined the spectral changes as we removed lignin by treatment with acidic bleach (Carpita, 1984). This extraction resulted in a decrease in the ferulic acid and lignin-related peaks at 1697, 1633, 1603, and 1171 cm^−1^. The cellulose/hemicellulose strongest peaks at 1122 and 1093 cm^−1^ became the major spectral features (Fig. 7A, iii). Raman mapping of the peak intensity at 1630 cm^−1^, which represents ferulic acid, revealed the familiar spotted pattern, with low intensities marking the spots (Fig. 7B). After treatment with NaOH solution, the pattern at the ITCW was still visible, although less clear (Fig. 7C). The acidic bleach treatment removed the Raman pattern completely (Fig. 7D). We could not detect the pattern by plotting other peaks. In correlation, the blue and green autofluorescence pattern was also weaker after NaOH treatment and undetectable after acidic bleach treatment. Moreover, the acidic bleach treatment also removed the bright SEM-BSE structures (Supplementary Fig. S2).

**Fig. 8.**
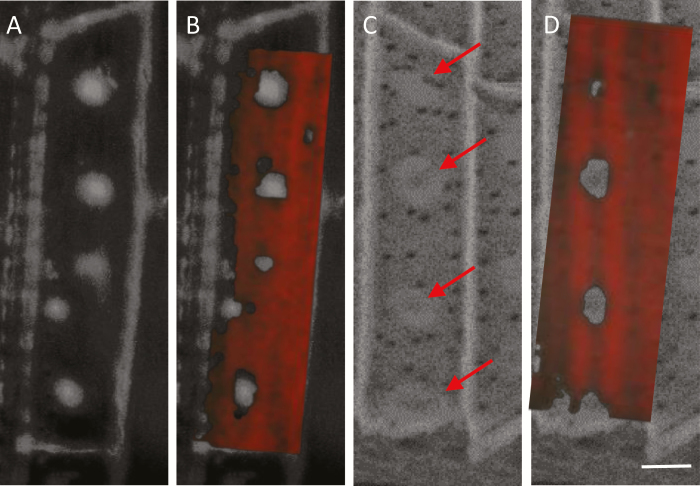
Spatial correlation between autofluorescence, Raman, and SEM of the ITCW of the endodermis at zone iv, imaged in roots of Si– sorghum seedlings after removal of the cortical tissues. (A) Green autofluorescence of the ITCW of a single endodermis cell. The radial cell walls are seen as a frame to the spots. (B) Intensity of the Raman scattering at 1630 cm^−1^ of the same cell is plotted in red, on top of the fluorescence map. (C) SEM-BSE micrograph showing a pattern of bright structures within the ITCW (arrows). (D) Intensity of the Raman scattering at 1630 cm^−1^ of the same cell is plotted in red, on top of the SEM-BSE micrograph. Low Raman intensities are co-localized to both the autofluorescent green spots and the bright structures visible in SEM-BSE, suggesting that the SEM-BSE and fluorescence patterns are also co-localized. Scale bar=5 µm.

To link the spectral features to the autofluorescent spots and the structures visualized by SEM-BSE, we measured the same endodermis cell by both autofluorescence and Raman (Fig. 8A, B), and by SEM-BSE and Raman (Fig. 8C, D). Plotting the ferulic acid peak at 1630 cm^−1^, we mapped its lowest relative intensities to the locations of high green autofluorescence and to high SEM-BSE brightness. These results confirm that the spotted pattern observed by the three different methods co-localize (Fig. 8). Collectively, this observation suggests that the same material producing the spotted pattern is detected by the three methods of microscopy and is removed by acidic bleach. We propose that the spots are made of a lignin-related compound.

**Fig. 9.**
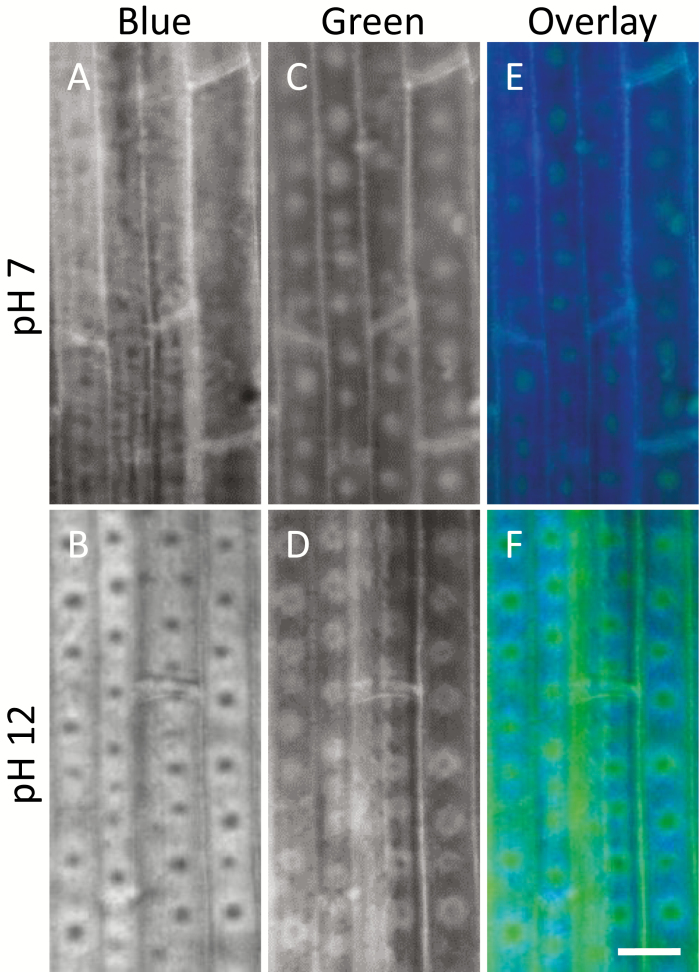
Changes in the autofluorescence of the ITCW of root endodermis at zone iii in Si– sorghum seedlings with changes in pH. (A) Under neutral pH, blue epifluorescence shows a pattern spots of low fluorescence at the ITCW. (B) Under high pH, the low-intensity spots are enhanced, indicating that the fluorescent cell wall contains unmodified lignin. (C) Green epifluorescence shows a pattern of spots with high fluorescence at the ITCW. (D) Under high pH, the green spots are extended, and their center loses its fluorescence. (E) Merged image of the green and blue epifluorescence at neutral pH demonstrates that in the ITCW the two signals do not overlap. (F) Merged image of the green and blue epifluorescence at high pH reveals a ‘bagel’ structure, with a dark center encircled by a green ring, which in turn is encircled by a blue ring. Scale bar=10 µm.

To characterize lignin modifications at the ITCW, we compared autofluorescence under neutral and basic conditions (Fig. 9). Unmodified lignin has blue autofluorescence, which is enhanced under basic pH, while modified lignin may fluoresce in green, depending on specific chemical modifications (Abraham *et al.*, 2018). Enhanced fluorescence and a shift to green fluorescence under high pH is a result of the ionization of lignin hydroxyl and/or carboxyl groups. However, if these groups are engaged in ester or ether bonds, the fluorescence is quenched (Meyer *et al.*, 2003). Blue autofluorescence of the Si– ITCW was enhanced when observed immediately after mounting in 1 M NaOH. In contrast, the spots were less fluorescent (Fig. 9A, B). This indicated a fraction of non-modified lignin surrounding the spots. The spots themselves presented green autofluorescence, which disappeared with high pH only at the center of the spots (Fig. 9C, D). Superimposing the green and blue epifluorescence signals, we found that the radial cell walls and some of the ITCW changed their fluorescence from blue at pH 7 to green at pH 12 (Fig. 9E, F). This indicated the presence of ester-bound ferulic acid with a phenolic hydroxyl that was free to ionize, possibly modifying hemicellulose (Harris and Hartley, 1976). Under high pH, the green spot itself showed a radial variation in autofluorescence: dark at the center, surrounded by a green ring, which in turn was surrounded by a blue ring.

**Fig. 10.**
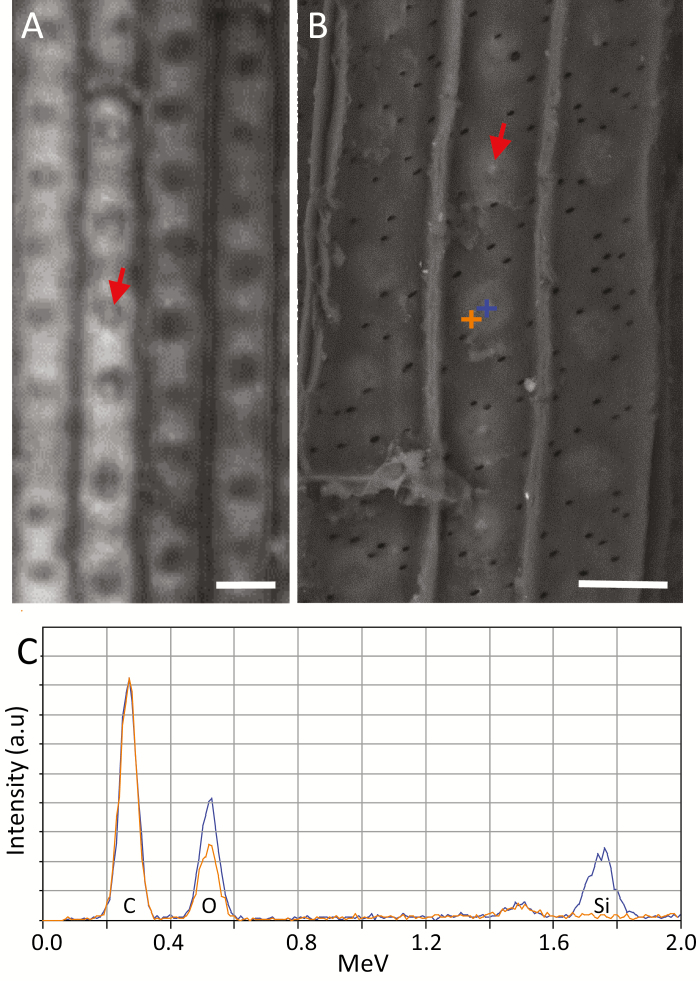
Imaging of the ITCW of Si– root endodermis in zone iv. (A) Blue autofluorescence at high pH reveals a bright dot (arrow) at the center of some of the low-fluorescence spots. (B) In the center of some of the bright structures observable by SEM-BSE, a dot (arrow) brighter than the organic background can be identified. SEM-EDX spectra were collected at the + signs. (C) The SEM-EDX spectrum at the dot (blue +) suggested that silica aggregated in this location. The SEM-EDX spectrum adjacent to the dot (orange +) indicates that Si content was below the limit of detection. Scale bars=10 μm. This figure is available in color at *JXB* online.

Interestingly, the very center of the green spots, which did not fluoresce under high pH in most spots, did fluoresce in blue in rare cases. Blue fluorescence was observable as a central dot under high pH, in the very mature parts of some of the roots (Fig. 10A). In the same regions scanned by SEM-BSE, we could also identify a bright dot in the middle of some of the bright structures (Fig. 10B). These dots were identified by SEM-EDX as silica (Fig. 10C). We explain the deposition of silica in Si– roots as the result of a very minute amount of silicic acid as a contaminant in the hydroponic solution. This finding assigns a role of silica nucleation to the lignin-based spots at the ITCW.

## Discussion

### The pattern of silica deposition is strictly controlled

Our results show that the tertiary thickening of the ITCW includes the deposition of modified lignin and silica in a pattern of spots. Lignin patterning can be achieved by patterning the binding of cell wall NADPH oxidases and peroxidases to the cell membrane through scaffold proteins, which creates local production of hydrogen peroxide (Lee *et al.*, 2013). Laccases that produce radicals of monolignols can be linked specifically to secondary cell walls, possibly through their glycosyl modifications (Yi Chou *et al.*, 2018). They can also create localized spots of lignin polymerization (Schuetz *et al.*, 2014). The deposition of lignin at the ITCW starts together with the formation of the silica aggregates in Si+ roots (Soukup *et al.*, 2017). In Si– roots, we detected a micron-scale patterning of lignin types that exhibit variation in their autofluorescence (Fig. 9). This highly regulated process must be actively controlled by living endodermis cells, in agreement with our previous results showing that the aggregation of silica depends on metabolic energy (Soukup *et al.*, 2019). The spotted pattern starts to develop at ~4 cm from the root tip. In this region lateral roots are emerging, and the major role assigned to the endodermis is mechanical (Geldner, 2013). Silica and lignin may strengthen the ITCW. However, the biological role of the complex lignification–silicification patterning is unknown. The first spots, which form in zone ii (Fig. 3), are located distantly from one another, with a gap of about twice the width of the cell (Fig. 4). In zone iii, new spots appear between the older ones, creating a weak pattern of alternating larger and smaller aggregates. This variation diminishes as the aggregates complete their growth in zone iv. Under Si depletion, the appearance of the spotted pattern is delayed. This is in agreement with the delayed development of the endodermis in Si– roots of rice and maize (Fleck *et al.*, 2011; Lukačová *et al.*, 2013). In sorghum, the spots are established in Si– roots only in zone iii, where they assume a spatial distribution similar to the aggregates in zone iii of Si+ roots (Fig. 6). Our observations show that the root tissue prepares itself for the deposition of silica, and there are no silica deposits in young tissues (Fig. 3). In addition, in Si– roots that are exposed to silicic acid, a gradient of aggregate sizes can be observed (Soukup *et al.*, 2017). The largest aggregates develop in zones iii and iv, and smaller aggregates are located in older tissues, close to the root–shoot junction. This suggests that there is a region where the deposition is most effective, in correlation with the formation of the tertiary cell wall. This is suggested to be the ASZ. The whole process occurs within hours, suggesting that transcription and translation of the relevant genes has already occurred in the younger parts of the root.

### The spots are possibly made of densely packed lignin

The greater brightness of the non-silicified cell wall at the putative silica deposition spots observed by SEM-BSE (Fig. 6) could be related to a higher topography or density of the cell wall. However, the topography is not pronounced, as can be seen in root sections (Fig. 1C, Supplementary Fig. S1; see also Sangster and Parry, 1976; Lux *et al.*, 2003; Soukup *et al.*, 2017). Similarly, lignin, cellulose, and hemicellulose have the same dry density (~1.5 g ml^–1^; Raven *et al.*, 2005) and atomic fraction of C and O to H (with a general formula of C_5–6_H_10–12_O_4–7_). Nonetheless, since the bright spots seen by SEM-BSE were eliminated with the removal of lignin (Supplementary Fig. S2), we conclude that they are made of lignin that was packed in a denser manner than the other cell wall constituents. A possible structure could be the stacking of aromatic rings of lignin monomers. This organization may also cause a red shift of the autofluorescence of unmodified lignin, explaining the distinct green autofluorescence we detected in the dense cell wall spots (Fig. 6).

Careful autofluorescence analysis located ferulic acid modifying hemicellulose in the radial and bulk inner tangential cell walls. Fig. 9 shows that in these regions there was a shift from blue to green autofluorescence with a pH change from 7 to 12. This indicated the presence of free hydroxyl groups on ferulic acid moieties, typically bound to hemicellulose (Harris and Hartley, 1976). However, the blue to green shift was not observed in the silica nucleating spots. The absence of ester-bound ferulic acid at the center of the spots was supported by chemically removing hemicellulose with NaOH. We could still identify the green fluorescent spots in root tissue subjected to this treatment. In addition, mapping of the ferulic acid Raman peak revealed that the spotted pattern was still present after the removal of hemicellulose by NaOH (Fig. 7C). This, too, indicated that ferulic acid bound to hemicellulose is not localized uniquely to the silica-nucleating spots. Instead, the green fluorescence of the spot centers disappeared under high pH (Fig. 9D). This may be explained by the disruption of densely packed lignin.

### The aggregates form layer by layer only at their base

**Fig. 11.**
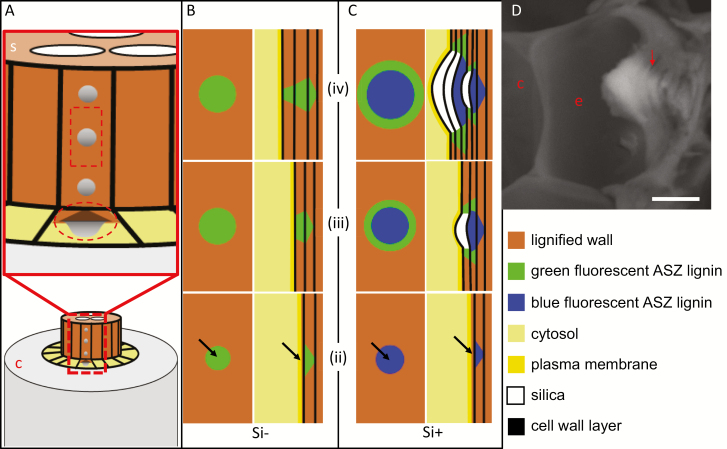
Suggested model for the formation of silica aggregates in sorghum root endodermis. (A) Scheme of a root section, showing the cortex (c) in gray, the stele (s) in brown, and the endodermis cell layer in yellow. The upper diagram shows a magnified view of the stele and endodermis, with one cell exhibiting silica aggregates (dark gray), viewed from the cytoplasm of the endodermis cell (marked by a rectangle) and in a cross section (marked by a circle). (B, C) Schemes showing a suggested developmental sequence of the endodermis ITCW in zones ii (bottom), iii (middle), and iv (top) in Si– (B) and Si+ (C) roots. The left panels show a longitudinal view, similar to the rectangle marked in (A). The right panels show cross sections, similar to the circle marked in (A). Arrows in zone ii indicate a putative initiation center of ASZ lignin. (D) SEM-BSE image of a cross section of a fresh adventitious root of sorghum grown in soil, showing an endodermis cell (e) and adjacent cortex cell (c). The developed silica aggregate in the ITCW (arrow) shows cell wall layers (darker areas). Scale bar=5 μm.

Our observations are in contrast to our previous findings showing that root silica extracted by sulfuric acid contains hemicellulose and ferulic acid (Soukup *et al.*, 2017). We can explain this by separating the nucleation process from the growth of a silica aggregate that may capture ferulic acid-bound hemicellulose. We suggest a sequence of events that may produce silica aggregates at the endodermis ITCW in sorghum (Fig. 11). According to our model, the endodermis cell produces a local spot at its cell wall, where a unique lignin polymer is synthesized. We term this polymer the ASZ lignin. The putative ASZ lignin has extreme affinity for silicic acid and the ability to catalyze the condensation of silicic acid to silica even under residual concentrations of silicic acid (Fig. 10). Under Si starvation, the ASZ lignin is incorporated into the cell wall, making it fluorescently green (Fig. 11B). In the presence of silicic acid, the ASZ lignin monomers/oligomers cross-link to the cell wall and at the same time interact with the apoplastic silicic acid. In this form, the ASZ lignin is fluorescently blue (Fig. 11C, bottom panel). As the polymerization proceeds in zone iii (Fig. 11C, middle panel), some ASZ lignin is captured in the growing mineral before cross-linking to the cell wall. Close to the location of ASZ lignin formation, silica polymerizes very rapidly, incorporating only little ASZ lignin and cell wall polymers. Some of the ASZ lignin units that are not interacting with silicic acid (possibly due to a lack of free acid) diffuse to the rim of the aggregate and create cross-links between the cell wall polymers, maybe in a similar fashion to the cross-links created by ferulic acid (Hatfield *et al*., 2016). This non-silicified ASZ lignin may be the source of the green fluorescence at the periphery of the aggregate, similar to the green spots in Si– roots. Binding to the cell wall changes these lignin units and they lose their ability to polymerize silicic acid. The final form of the aggregate includes a layer-by-layer deposition of cell wall and silica at its base and margins, and mostly silica at its top (Fig. 11C, top panel). A cross-sectional SEM-BSE image of an aggregate embedded in the endodermis ITCW shows the layers of (mostly) cell wall at the rim of the aggregate and (mostly) silica at its center (Fig. 11D).

### Silica coupled to lignin polymerization may affect cell wall oxidative levels

The variation in autofluorescence coupled with silicic acid availability and silica deposition indicates that Si influences the cell wall chemistry. Specifically, our work demonstrates a strong effect of Si on lignin structure or composition. Control over lignin composition and its incorporation into the cell wall are major aims in developing crops for biofuels and fodder. The polymerization of lignin monomers into the functional polymer occurs in the cell wall, as a result of the activity of reactive oxygen species (ROS) (Wang *et al.*, 2013). The ROS are patterned by the localization of ROS-producing and manipulating enzymes on the cell membrane. Lignin monomers are suggested to be added to the growing polymer in a combinatorial manner, and the monolignols are suggested to diffuse quickly through the cell membrane and in the cell wall. This model suggests a uniform and random incorporation of varied monolignols into the cell wall. There are examples of variation in the composition of cell wall-lignin with time (Perkins *et al.*, 2019). Our data demonstrate variation in lignin on a size scale of microns within a single cell wall layer. We found that at the aggregate formation site ASZ lignin accumulated, surrounded by non-modified lignin, which was linked to hemicellulose through ferulic acid (Figs 5 and 7). This requires a mechanism to control highly local spatial variations in monolignols. Our results support the existence of putative monolignol active transporters bound at specific locations on the cell membrane. ATP-binding cassette (ABC) transporters that may actively export specific monolignols (Perkins *et al.*, 2019) could be involved in the formation of root silica aggregates.

Silicic acid and silica availability correlate with a reduction of ROS activity in various systems (e.g. Liang *et al.*, 2003; Gong *et al.*, 2005; Fleck *et al.*, 2011; Mehrabanjoubani *et al.*, 2019). Our results suggest that Si also affects the chemistry of lignin. Further molecular research is needed to test whether the formation of silica in the cell wall affects ROS balance and results in changes in the level of oxidative stress in tissues. The control over the localized formation of silica aggregates uncovered in this work indicates an important biological role for silica in roots. Understanding silica deposition at the root is a key step towards understanding the role of Si in plant biology.

## Supplementary data

Supplementary data are available at *JXB* online.

Fig. S1. SEM-BSE micrograph of a cross-section of a Si– root stripped of its cortical tissues.

Fig. S2. SEM-BSE micrograph of the ITCW of Si root, treated with acidic bleach.

eraa127_suppl_Supplementary_Material

## Abbreviations:

ASZ: active silicification zone
BSE: back-scattered electrons
EDX: energy-dispersive X-ray
ITCW: inner tangential cell wall
ROS: reactive oxygen species
