# Supplementary material for: Unique lignin modifications pattern the nucleation of silica in sorghum endodermis
Source: J Exp Bot. 2020 Mar 9;71(21):6818–29. doi: 10.1093/jxb/eraa127 (PMC7709913; doi:10.1093/jxb/eraa127)
Supplement: eraa127_suppl_Supplementary_Material [file eraa127_suppl_supplementary_material.pdf]

Supplementary Material

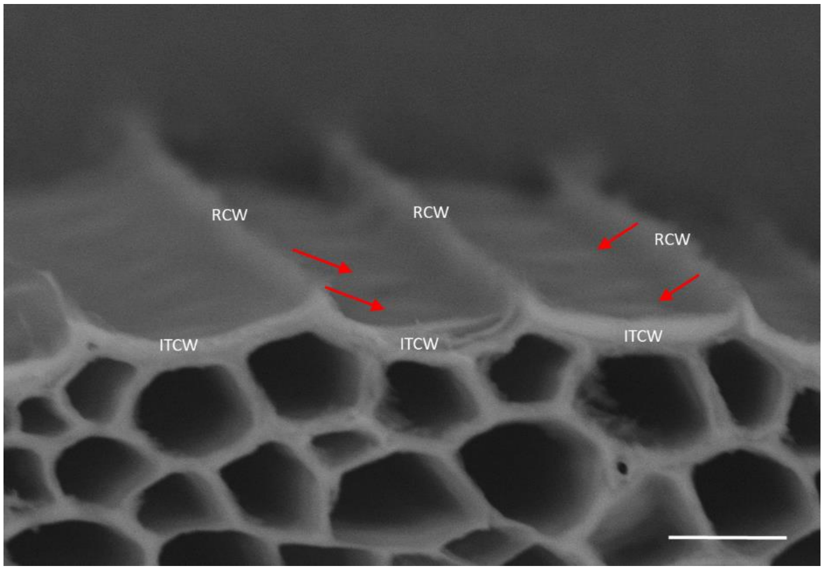

Figure S1. SEM micrograph of a cross section of a Si- root stripped of its cortical tissues. This is a side view of the endodermis ITCW showing the bright structures along the walls (arrows). The structures appear to have little or no topography. ITCW=inter tangential cell wall, RCW=radial cell wall. Scale bar represents 10 μm.

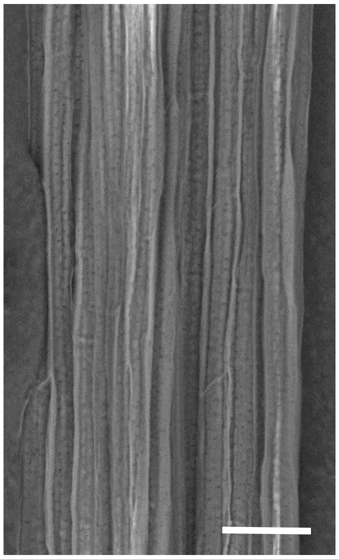

Figure S2. SEM micrograph of the ITCW of Si- root, treated with acidic bleach. A sorghum root from Zone iii was stripped of its cortical tissues and treated with acidified bleach for 24 H. No bright structures could be detected along the endodermal ITCW. Scale bar represents 50 μm.
